# Exploring Social Media to Understand Perceptions of Milk Quality among Farmers, Processors, and Citizen-Consumers

**DOI:** 10.3390/foods13162526

**Published:** 2024-08-13

**Authors:** Michele Paleologo, Greta Castellini, Albino Claudio Bosio, Michele Fontana, Guendalina Graffigna

**Affiliations:** 1 EngageMinds HUB—Consumer, Food & Health Engagement Research Center, Università Cattolica del Sacro Cuore, 20123 Milan, Italy; michele.paleologo@unicatt.it (M.P.); claudio.bosio@unicatt.it (A.C.B.); guendalina.graffigna@unicatt.it (G.G.); 2 Faculty of Agriculture, Food and Environmental Sciences, Università Cattolica del Sacro Cuore, Via Bissolati, 74, 26100 Cremona, Italy; 3 Department of Psychology, Università Cattolica del Sacro Cuore, Largo Agostino Gemelli, 1, 20123 Milan, Italy; 4 IRCAF (Invernizzi Reference Center on Agri-Food), Campus Santa Monica, Via Bissolati, 74, 26100 Cremona, Italy; 5 Department of Informatics, System and Communication (DISCo), University of Milano-Bicocca, Piazza dell’Ateneo Nuovo, 1, 20126 Milan, Italy; m.fontana36@campus.unimib.it

**Keywords:** social media, milk quality, farmer, consumer, processors, sentiment analysis, content analysis, consumer psychology

## Abstract

Milk consumption is crucial for a balanced diet, yet recent trends indicate a decline, especially in Italy. A significant factor in this decline is the altered perception of milk quality among consumers, which has created a communication gap between them and other stakeholders. This study aimed to explore the discourse on social media and sentiment towards the concept of milk quality among consumers, farmers, and processors. The research adopted social media analysis to examine online-community messages. A sample of 19,906 Italian comments and posts mentioning keywords “milk”, “quality”, “cow”, and “vaccine” was collected and categorized using term-frequency analysis, correspondence analysis, and sentiment analysis. Results highlighted gaps in perceptions of milk quality: farmers focused on economic issues, consumers on animal welfare and health, and processors on lactose content. For farmers, almost all comments were negative, while for processors, nearly all comments were positive. Consumers presented a more mixed picture. This work contributes to the literature by expanding research on milk quality, using social media as a source of information. The findings suggest that enhancing communication and understanding among these groups could lead to more effective strategies for addressing consumer concerns, potentially reversing the decline in milk consumption.

## 1. Introduction

The consumption of milk has long been recognized as an essential component of a balanced diet, as milk provides vital nutrients such as calcium, protein, and vitamins [1,2]. However, recent trends indicate a decline in milk consumption [3]. Specifically, data reveal a 2% reduction in milk consumption within the EU between 2013 and 2018, with projections indicating a continued decline [4]. This trend is particularly pronounced in Italy. In the past half-decade, Italian households have experienced a 7% reduction in milk purchases, primarily impacting fresh milk, followed by long-life milk (ultra-high-temperature-treated (UHT) milk) between 2016 and 2021 [5,6]. Despite initial signs of UHT milk consumption rebounding during the COVID-19 pandemic, consumption resumed its downward trajectory in 2020/2021 [7]. These data, gathered from nationwide surveys, indicate a notable shift in consumer preferences away from traditional dairy products. Furthermore, market research conducted by Euromonitor International (2022) [8] supports this observation, indicating a steady decrease in the sales of conventional milk products across various retail channels in Italy. The data suggest a growing consumer inclination towards plant-based milk alternatives, such as almond milk, soy milk, and oat milk, raising concerns about the trend’s implications for public health and dairy-industry sustainability.

One of the factors contributing to this decline is the changed perception of milk quality among citizen-consumers, which has resulted in a significant cultural and communication gap among citizen-consumers, dairy producers, and processors [4]. A recent systematic literature review highlighted how consumers, farmers, and processors have different representations of milk quality, leading to a disconnect between citizen-consumer expectations and industry practices [9]. Indeed, while farmers and processors demonstrated a comparable understanding of milk quality that involves emphasizing technical criteria, citizen-consumers, on the contrary, tended to have simpler and more subjective opinions that were difficult to measure quantitatively. Dairy experts, including farmers and processing specialists, emphasized that milk quality is ensured through careful attention to animal welfare, which involves practices such as disease monitoring, pathogen detection through milk testing, appropriate treatment methods, and effective mastitis-management strategies. Conversely, citizen-consumers argued that milk quality is primarily linked to the well-being of animals, emphasizing their natural behaviours such as grazing and consuming grass. Moreover, while experts focused on the nutritional value of milk, considering factors like energy, protein, and calcium content, citizen-consumers prioritized the absence of additives and the naturalness of the product when defining milk quality. These findings align with prior research indicating that citizen-consumers are placing a growing emphasis on scrutinizing nutritional content, preferring products without harmful additives, and assessing the overall health and environmental implications of their consumption choices [10]. Recent studies have underscored the impact of health and animal-welfare concerns on citizen-consumer attitudes toward milk, affecting consumption behaviours [11]. Moreover, sustainability and ethical considerations have become increasingly influential in shaping perceptions of food quality, with citizen-consumers prioritizing environmentally sustainable production methods, fair-trade principles, and animal-welfare standards [12].

However, these studies were conducted using secondary data or cross-sectional surveys. While valuable, these approaches may overlook nuanced insights that can be gleaned from more spontaneous and direct forms of communication, such as social media platforms [13]. The utilization of social media platforms for generating and sharing information and opinions represents a valuable method for understanding people’s perceptions and sentiments across various sectors of society [14,15]. Increasingly, people are turning to the internet for diverse activities, ranging from information retrieval to online transactions. Social media platforms, in particular, serve as virtual hubs for exchanging opinions and information, making them rich sources of citizen-consumers’ insights [16] and emotions [17]. Moreover, social media platforms provide opportunities to gain valuable insights into people’s perceptions of product attributes [18]. Additionally, people are often more inclined to express their opinions about products on social media platforms than through traditional surveys [19]. By analysing the content of social media, it is possible to glean insights into people’s attitudes toward specific issues or products. Social media marketing operates on the premise that social media content is a dialogue initiated by citizen-consumers, audiences, or businesses [20]. The interactive nature of communication on social media enables companies and citizen-consumers to learn from each other about the practical use of products [21]. Social media platforms serve as arenas for information exchange, communication, and engagement [22]. Overall, leveraging social media as a research tool offers a dynamic and comprehensive approach to understanding the multifaceted dimensions of product attributes, comparing different points of view. Although this methodology has been used to investigate people’s perceptions of different food products [13,23], to the best of our knowledge, there have been no studies conducted on social media to investigate the perspectives of citizen-consumers, processors, and farmers regarding the concept of milk quality.

Based on these premises, it is crucial for the dairy industry to understand and explore the societal perspective on milk quality, as emphasized by the Food and Agriculture Organization of the United Nations (FAO) [24]. In particular, it is essential to examine the new perceptions and quality characteristics of citizen-consumers regarding milk quality and determine if they align with those of experts such as farmers and processors, utilizing diverse research methods including social media analysis. This understanding is vital for product development and the creation of marketing strategies that address the constantly evolving preferences and demands of citizen-consumers [4,25].

To bridge these knowledge gaps, the aim of this study is to explore the discourse on social media and sentiment towards the concept of milk quality among consumers, farmers, and processors. In particular, the objectives of this study are as follows: (a) to shed light on how citizen-consumers, farmers, and processors perceive and discuss milk quality through the analysis of spontaneous comments and discussions on social media platforms such as Facebook and YouTube; (b) to investigate the disparities and parallels in how milk quality is perceived among these actors. These platforms were chosen because they are where Italians most frequently comment on such topics. While platforms like Twitter and Instagram were considered, Facebook and YouTube were deemed more suitable due to their higher usage rates among Italians—77% for Facebook compared to 25% for Twitter—which make them more representative of the population [26].

## 2. Materials and Methods

**Figure 1:**
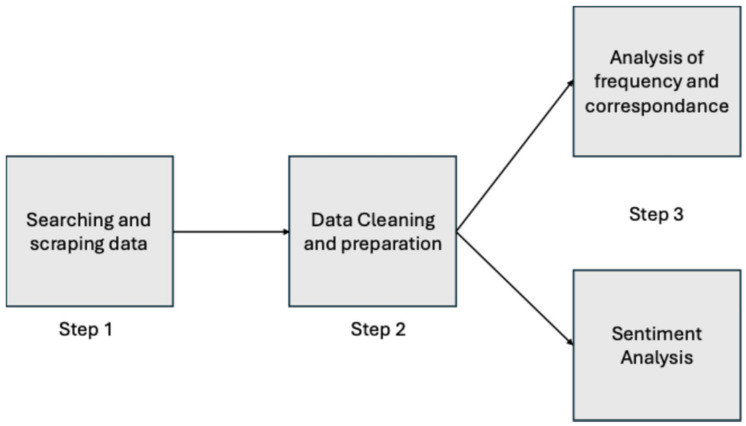
Data collection and analysis.

Data collection and analysis were carried out in several steps, as illustrated in Figure 1. The following sections will explain the individual search steps in detail.

### 2.1. Data Collection

A search without time restrictions was conducted on the social networks Facebook and YouTube to collect comments in Italian related to milk quality via the search strings “Latte”, “Qualità”, “Mucca”, or “Vaccino” (which translate to “Milk”, “Quality”, “Cow”, and “Vaccine”, respectively). The comments were then divided according to whether they were written by a Consumer, Farmer, or Processor as follows:

- Farmers: Comments were sourced from sector-specific pages and groups with over 5000 members to ensure the user was a farmer.
- Processors: Comments were gathered from posts and comments on the pages belonging to the main Italian milk brands.
- Consumers: Comments were derived from posts on major consumer-association pages.

### 2.2. Data Cleaning and Preparation

The dataset contained four attributes: text, time, role, and link. Prior to analysis, the text data underwent several preprocessing steps. First, comments with fewer than five words were removed as they were considered too brief to be informative. Next, any artifacts generated during the data-scraping process were eliminated and embedded links were removed. Artifacts, in this context, refer to any unwanted or irrelevant data that may have been accidentally collected during the scraping process. Stopwords, which are common words like “and”, “the”, and “is” that typically do not carry significant meaning, were then removed using a custom function created by the authors; the list of stopwords used is available upon request. Lastly, the text was lemmatized using the Simplelemma library [27], which is designed to convert words to their base or dictionary forms, known as lemmas. This standardization process, known as lemmatization, helps to reduce the complexity of the text data by ensuring that different forms of the same word (e.g., “running”, “ran”, and “runs”) are treated as a single term (“run”). This step was crucial for facilitating meaningful analysis by improving the consistency and comparability of the textual data.

### 2.3. Frequency and Correspondence Analysis

A frequency analysis of the most common lemmas in the comments was then conducted and was followed by a correspondence analysis using QDA Miner (version 4.0; Provalis Research, Montreal, QC, Canada), a qualitative data-analysis program that facilitates the organization, coding, and analysis of textual and visual data [28,29]. Correspondence analysis is a statistical technique used to identify and visualize relationships between categorical variables in a dataset. It allows us to detect patterns and associations between rows and columns of a contingency table, making it easier to interpret complex data. We used correspondence analysis to uncover and illustrate the relationships between different lemmas and the roles of the users, providing deeper insights into the underlying patterns in the dataset.

### 2.4. Sentiment Analysis

Sentiment analysis was subsequently performed on the comments using the “feel-it-Italian-sentiment” library, an open-source tool tailored to the Italian language and fine-tuned from the UmBERTo model. Sentiment analysis is a technique used to determine the emotional tone behind a series of words, typically classifying text as positive, negative, or neutral. This analysis helps in understanding the overall sentiment or opinion expressed in the text. The “feel-it-Italian-sentiment” library was specifically chosen because it is designed for the Italian language, ensuring more accurate sentiment classification for the dataset. By applying this model, we were able to assign each comment a sentiment score, which can be positive or negative, thus allowing us to gauge the emotional tone of the comments and identify prevalent sentiments within the data.

## 3. Results

### 3.1. Description of the Text Units

The scraping procedure resulted in a total of 19,906 text units composed of comments and posts. After the cleaning procedure, the total number of text units dropped to 15,508. Most comments of the had been written by consumers (*n* = 13,410, 87%), followed by processors (*n* = 1218, 8%) and farmers (*n* = 850, 5%). Farmers posted their discussions on milk quality mainly on Facebook, specifically on pages dedicated to industry magazines and within farmer-specific groups. Processors’ comments on milk quality were found on their company pages on Facebook. Consumers’ comments appeared on Facebook on consumer-association pages, on pages belonging to large dairy companies, and in response to news from major news outlets. Additionally, consumer discussions on milk quality were also identified on YouTube in the form of videos or comments on videos.

### 3.2. Trend over Time

The comments retrieved have a date range from 2012 to 2023, but less than 1% of the comments from all three types of actors were made before 2014.

**Figure 2:**
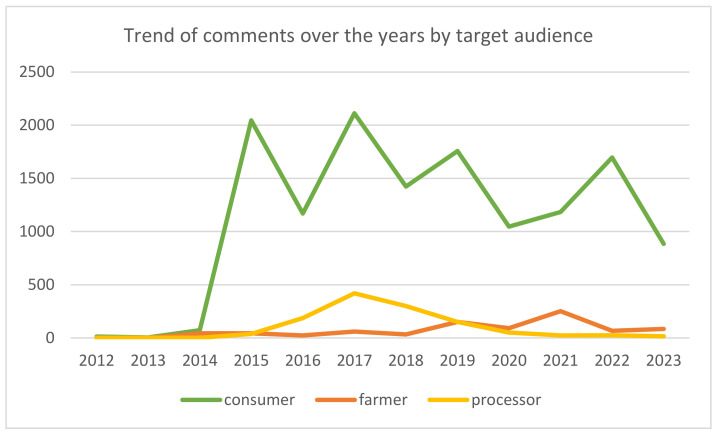
Trend chart of comments over the years.

2017 saw the greatest number of total comments (2592), followed by 2019 (2062), as can be seen in Figure 2. It is also interesting to note that the 2017 peak is evident for consumers and processors but not for farmers, whose main peak is in 2021, when almost 30% of the comments were made (251).

### 3.3. Analysis of Frequency

**Figure 3:**
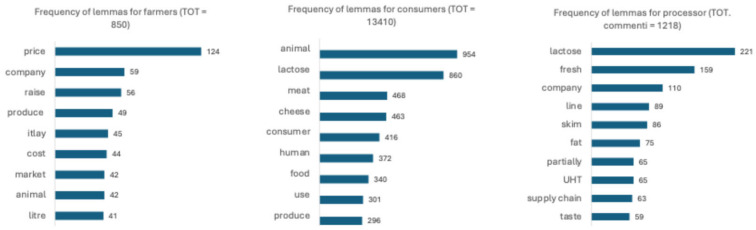
Lemmas related to milk quality.

The analysis of the frequencies of the lemmas per type of actor included in this study shows clear differences that can be traced back to different discourses. In fact, as shown in Figure 3, in the case of farmers, the three most-used words were “price”, “company”, and “raise”, while in the case of consumers, the most-used words were “animal”, “milk”, and “meat”; finally, the processors most often used the words “lactose”, “fresh”, and “company”.

In the case of farmers, the lemmas “prices”, “costs”, “increase”, “company”, and “production” were found in discussions of controversies over rising costs and the economic unsustainability of production, in a context in which a dichotomy between production quality and production quantity is often created. Furthermore, the Italian character of production emerged as a synonym for quality.

*«as long as the product you make is priced by others, it will always be a miserable business»*

*«if you start doing the accounts with the pen tomorrow morning you close the company»*

*«But are we sure that our milk is better, ‘more quality’? Do we have proof of what we have been repeating for years like a mantra or is it the usual way of saying it?»*

In the case of consumers, on the other hand, the issue of animal welfare was often prevalent; this topic, with different logic, was also linked to the issue of environmental sustainability. Furthermore, lactose, often defined as harmful to health, was a prevalent theme.

*«I hope it is true that you use Italian milk. We have the best and most excellent stuff and quality in the world and we go and get it from abroad, rotting our good stuff.»*

*«For almost a year now, I have given up cow’s milk in favour of lighter rice milk—it’s a different world. I feel more energetic and full of energy right from the morning, whereas before I always had a feeling of drowsiness that accompanied me in the first few hours after waking up.»*

*«poor animals in whose hands...»*

Finally, in the case of processors, a mirroring effect can be observed whereby the topic of lactose was strongly re-emphasised as they promoted their lactose-free products through social communication; this topic was mentioned even more often than was the freshness of the milk, just as the Italian character of the milk is emphasised to define its quality.

*«Have you ever tried #xxx milk? It is particularly*

*suitable for people like you who are intolerant and want an easy-to-digest milk that contains less than 0.1% lactose. This way, you won’t have to give up the rich nutritional properties and taste of real milk!»*

*«xxx has never imported milk from China, nor does it intend to do so. In fact, our mission is to offer our consumers a high quality product. For the sake of correct information, we also tell you that China produces a very low quantity of milk, not even enough for its own needs, so it is absolutely unthinkable to believe the false news that has been circulating these days.»*

### 3.4. Correspondence Analysis

**Figure 4:**
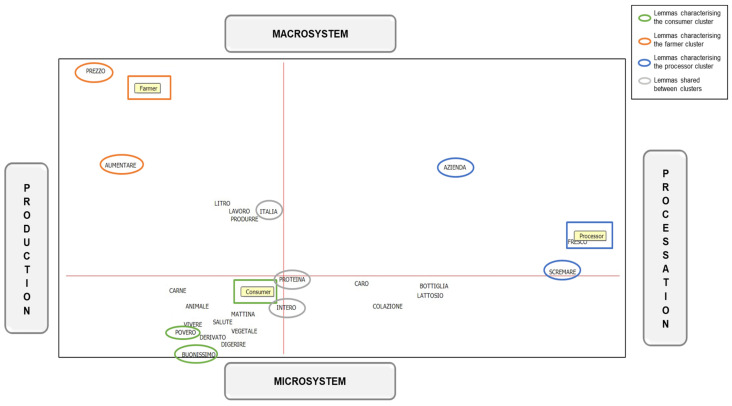
Cartesian plane resulting from correspondence analysis.

The correspondence analysis enabled the creation of a Cartesian plane to position the lemmas that most distinctly characterize the different roles, circled in Figure 4. In this plane, the x-axis can be considered to represent a continuum ranging from production (on the left), which involves livestock farming and the actual production of milk, to processing (on the right), encompassing the transformation phases before consumption. The y-axis, on the other hand, can be viewed as a continuum from the macrosystem (at the top) to the microsystem (at the bottom). The macrosystem refers to large-scale, overarching factors and entities, such as industry-wide practices and regulations, whereas the microsystem pertains to smaller-scale, localized elements, and particularly the product. On this level, it can be observed that farmers are in the macrosystem- and production-oriented quadrant, as their discourses were very much centred on price (“prezzo”) and increasing costs (“aumentare”). By contrast, consumers are in the microsystem- and production-oriented quadrant; in fact, differentiating this group are words referring to the product as “poor” (“povero”) and “good” (“buonissimo”). Finally, on the opposite side emerge the processors, whose discussions were particularly process-oriented but not especially polarised towards the macrosystem or micro-system and were characterised by the words “company” (“azienda”) and “skim” (“scremare”).

### 3.5. Sentiment Analysis

**Figure 5:**
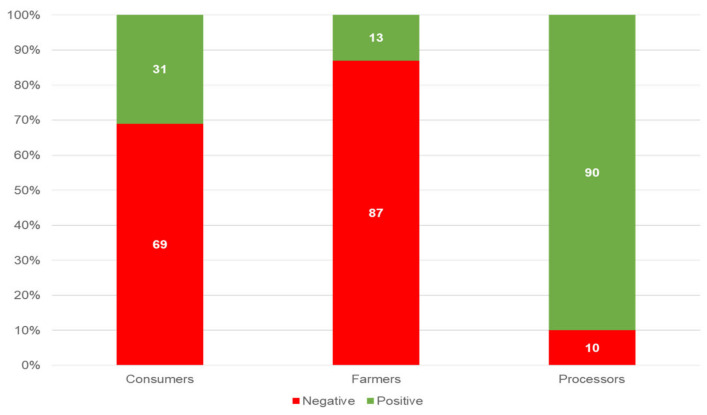
Sentiment scores of the three targets.

The sentiment analysis revealed significant differences among the three groups (Figure 5).

**Figure 6:**
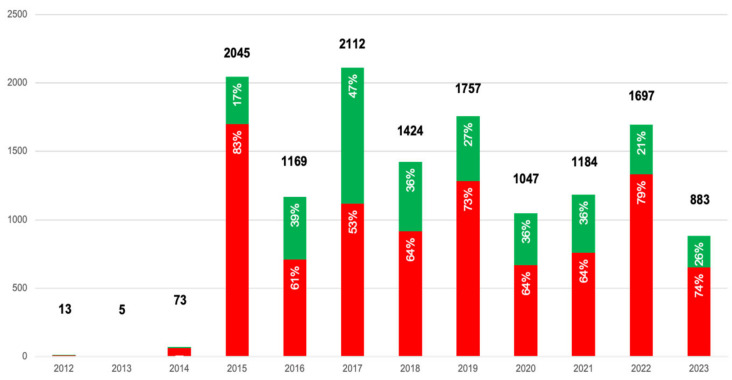
Trend chart of sentiment scores over the years.

For the farmers, almost all comments were negative (87%), while for the processors, the opposite was true, with nearly all comments being positive (90%). In contrast, the consumers presented a more mixed picture, with 69% of comments being negative and 31% being positive. It is noteworthy, moreover, that this proportion remained quite stable for processors and farmers, while for consumers, it changed over the years (Figure 6). In particular, looking at the three main peaks of comments, there is an evident difference in the proportion of positive sentiment among them, whereby in 2015, only 17% of the comments reflected a positive sentiment, while in 2017, almost half (47%) did so, and in 2019, about a third (27%) did so.

## 4. Discussion

This research has provided a comprehensive and ecological snapshot of the actual spontaneous social discourses surrounding milk quality taking place online, shedding light on the underlying reasons behind the decline in milk consumption observed in Italy in recent years [3]. By analysing spontaneous comments on social media, we have gained valuable insights into the perspectives of consumers, farmers, and processors regarding milk quality.

The differentiation in platform usage among stakeholders aligns with findings from previous studies that emphasize how social media platforms serve as distinct communication channels for various food actors [30]. Farmers often utilize industry-specific groups and pages to discuss production-related issues, reflecting a need for targeted communication within their professional community [31]. Processors’ preference for company pages to promote their products and engage with customers echoes findings by Kao et al. 2016 [32], which suggest that processors use social media primarily for marketing and customer relations. Consumers’ widespread engagement on consumer-association pages and major news outlets parallels the observations by Samoggia et al. (2020) [13], indicating that these platforms are pivotal for raising awareness and discussing quality and ethical issues.

The peaks in discussion, notably in 2017, for consumers and processors, and in 2021, for farmers, can be placed in the context of the industry events or crises that typically drive online discourse. In those years, in fact, several articles were published in the most important Italian newspapers about the serious accusations levelled at some Italian dairy companies involved in false certifications that tried to cover up the presence of aflatoxins in milk [33] at levels above the legal limits. These temporal spikes underscore the reactive nature of online discussions, where significant industry events prompt increased dialogue.

Considering the topics on which different actors talked about milk quality, the results reveal distinct discourses among the stakeholders, with farmers focused on economic issues, consumers on animal welfare and health (milk origin and lactose-free product), and processors on product attributes like lactose content and freshness, mirroring the topics most important to consumers. The results concerning consumers are corroborated by past research that showed that how animal welfare significantly influences consumers’ hedonic and emotional reactions to milk, increasing the intention to buy it [10]. Moreover, the emphasis on the product’s origin highlights consumers’ preference for locally sourced milk, which is perceived to be of higher quality and safer, as well documented by Canavari et al. (2015) [34].

The importance given by farmers to issues concerning the economic aspects of their farms reflects the problems that farmers are experiencing in Italy. Recent research has shown that in 3411 Italian farms (54% of the sample), the farm’s net income is lower than the reference income for family work, jeopardising the survival of these farms [35].

Finally, processors took up the same themes common among consumers and leverage them to promote their products. An example of this is the increasing presence of advertisements and communications emphasising the production of lactose-free milk to foster a positive behavioural intention in consumers towards these products [36]. Communication by these actors is therefore not disinterested, but is, on the contrary, strategically oriented towards profit and sales.

Furthermore, these differences are emphasised by the results of the correspondence analysis. They showed that the contents of the discourses concerning milk quality within the different groups belong to distinct focus areas and operational scopes. Indeed, the farmers’ discourses about milk quality were more focused on production and macrosystem aspects related to economic issues; consumers’ topics were focused on production aspects related to milk, emphasising microsystem aspects, i.e., were more related to intrinsic product qualities; finally, processors are more focused on aspects of technical processing related to milk. This highlights how each of these targets talked not only about different topics concerning milk quality but also about different subject areas and social systems. This supports the framework proposed by Bronfenbrenner (1979) [37], wherein different actors operate within nested systems, influencing and being influenced by both micro- and macro-level factors.

The communication gap among these three actors is particularly evident from the differing sentiment scores. While processors attempted to address consumer concerns, their overwhelmingly positive sentiment contrasted sharply with the more negative sentiments expressed by farmers and consumers. This discrepancy suggests a misalignment in the communication strategies of processors, whose communication may not fully resonate with the critical and concerned tones of the other actors. Farmers, in particular, stand out as having the most negative sentiments, reflecting their frustrations with economic pressures and the perceived disconnect between their efforts and consumer expectations. This variation corroborates findings by Whitaker (2024) [38], who found that economic pressures often lead to negative sentiments among farmers, whereas positive sentiment among processors can be attributed to marketing efforts that highlight positive aspects of their products [39]. The fluctuating consumer sentiment over time is consistent with findings by Shayaa et al. (2018) [40], who observed that consumer sentiment can be highly volatile and influenced by external events and media reports. This suggests that fostering direct communication and discussions among these three groups could enhance mutual understanding, improve attitudes, and possibly lead to more aligned and effective production practices.

Finally, it is interesting to reflect on the value of research based on spontaneous discourse from social media, highlighting aspects that cannot be fully understood using other methodologies. In fact, a recent systematic literature review conducted by Castellini et al. (2023) [9], with the same objectives as this study, reported only partially overlapping results. In this study based on social media, topics related to the concept of milk quality seem to align more closely with the common discussions among the various stakeholders. For instance, economic concerns are almost universally present in spontaneous social media discussions among farmers, an aspect that does not emerge in the systematic literature review. However, these issues are well-documented and genuinely exist among Italian farmers [35]. This evidence emphasises the need for more research using social media to understand people’s real perceptions of and attitudes towards certain issues, especially if they emotionally involve the participants and may reveal a negative feeling that might be hidden in other research contexts.

Despite these strengths, the present study has some limitations. The study’s reliance on social media data may introduce sampling bias, as it primarily captures the opinions and sentiments of users active on these platforms, potentially excluding those who are not engaged online. Moreover, the findings may not be fully representative of the broader population due to the differential qualities of social media users and their demographics, which could skew the results. Finally, due to the breadth of data sources and topics covered, the study may not delve deeply enough into specific aspects of discourse on milk quality, potentially overlooking deeper insights.

## 5. Conclusions

In conclusion, our study highlights significant gaps and areas of alignment in perceptions of milk quality among consumers, farmers, and processors. The findings have practical applications, suggesting that enhancing communication and understanding among these groups could lead to more effective strategies for addressing consumer concerns, thereby potentially reversing the declining trend in milk consumption. By focusing on the real-world issues discussed online, stakeholders can develop more targeted and relevant marketing and production strategies that better meet the evolving demands of today’s consumers.

The use of social media analysis offers a novel approach to understanding consumer perceptions in a more dynamic and immediate context. Future research should expand this methodology to other regions and food products to validate and extend these findings. Additionally, longitudinal studies could provide deeper insights into how these perceptions evolve over time and in response to industry changes. For consumers, this study highlights the importance of their role in shaping industry practices through their expressed preferences and concerns. By voicing their opinions on social media, consumers can influence the dairy industry to adopt more transparent and animal-friendly practices that align with their values. Policymakers should take into account the diverse perceptions of milk quality when developing regulations and standards for the dairy industry. Policies that promote transparency, animal welfare, and the naturalness of dairy products could help bridge the gap between consumer expectations and industry practices. Furthermore, supporting educational campaigns that inform consumers about the technical aspects of milk quality might help align consumer perceptions with industry realities.

Future research could benefit from longitudinal studies to track evolving consumer, farmer, and processor perceptions of milk quality, considering the influence of external factors. Cross-cultural comparisons would provide insights into regional variations in perceptions of milk quality. Finally, integrating social media analysis with traditional research methods like surveys could corroborate those findings and offer deeper insights into stakeholder viewpoints.

## Data Availability

The original contributions presented in the study are included in the article, further inquiries can be directed to the corresponding author.
